# Supplementary material for: PTENβ is an alternatively translated isoform of PTEN that regulates rDNA transcription
Source: Nat Commun. 2017 Mar 23;8:14771. doi: 10.1038/ncomms14771 (PMC5376652; doi:10.1038/ncomms14771)
Supplement: Supplementary Information — Supplementary Figures, Supplementary Tables and Supplementary References [file ncomms14771-s1.pdf]

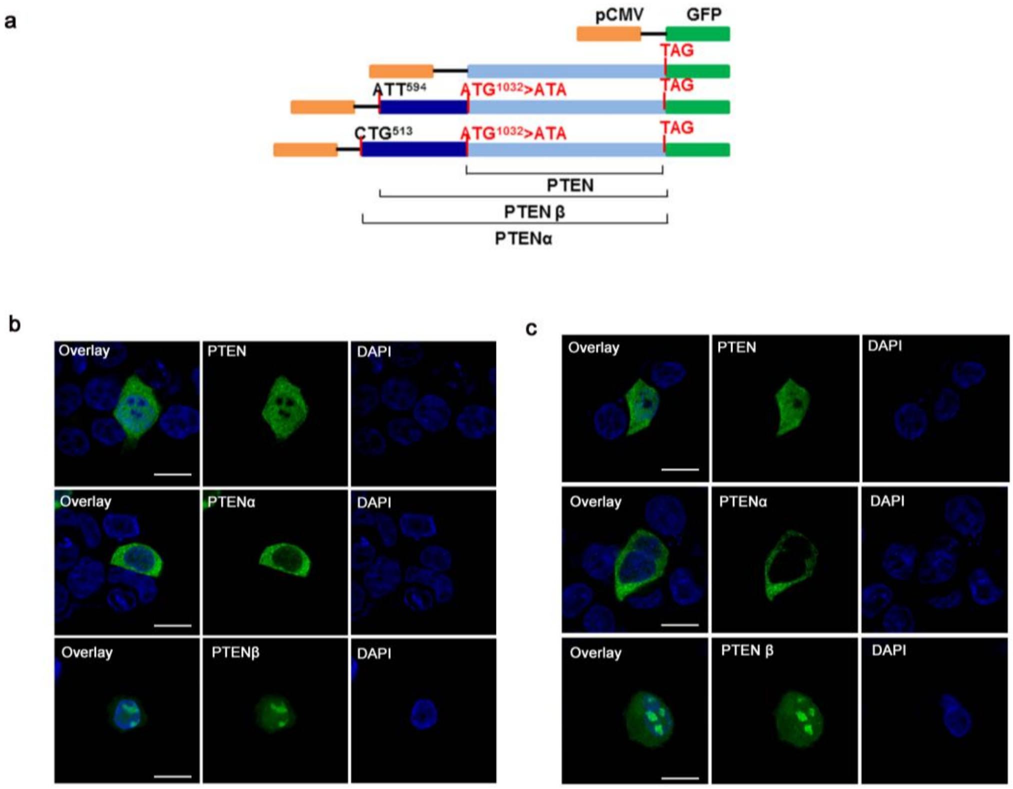

Supplementary Figure 1. Validation of the subcellular localization of PTENβ in the nucleolus.

(**a**) Differing sets of PTEN, PTENα and PTENβ constructs. A TAG triplet was inserted in the C-terminus of PTEN, PTENα and PTENβ sequences in constructs as indicated in Figure 4a, in order to abolish expression of GFP tag.

(**b,c**) Subcellular localization of exogenous PTENα, PTENβ or PTEN without tag. Constructs in (**a**) were introduced into Hela PTEN null cells. Twenty-four hours after transfection, cells were stained with DAPI and a PTEN antibody (ABM-2502) (**b**) or PTEN antibody (sc-7974) (**c**), followed by imaging with confocal microscopy. The scale bars represent 10μm.

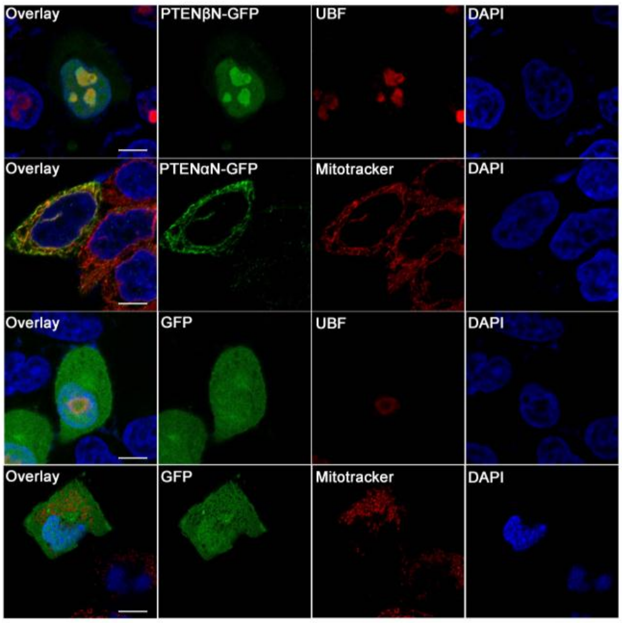

Supplementary Figure 2. The N-terminal extended sequences are solely responsible for localization of PTENα on mitochondria and accumulation of PTENβ in the nucleolus.

Plasmids expressing N-terminal extended sequences of PTENα (AA 1-173) and PTENβ (AA 1-146) were constructed with a C-terminal GFP tag. The indicated plasmids were introduced into Hela PTEN null cells, followed by imaging with confocal microscopy. UBF was used as a nucleolar marker and MitoTracker was used to label mitochondria. GFP tagged mock plasmids were transfected as a negative control. The scale bars represent 5μm.

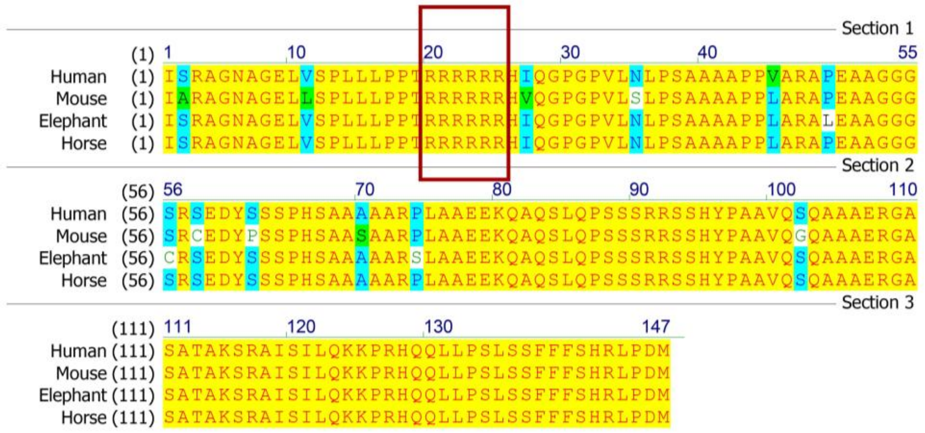

Supplementary Figure 3. PTENβ is evolutionarily conserved.

Clustal-W alignment of the N-terminal extended sequence of Human PTENβ with the genomic sequence from Mouse (Mouse Dec. 2011(GRCm38/mm10)), Elephant (Elephant Jul. 2009 (Broad/loxAfr3)) and Horse (Sept 2007(Broad/equCab2)) obtained from the UCSC genome browser (http://genome.ucsc.edu/). The conserved sequences are highlighted in yellow. The poly-arginine sequence in the N-terminal extended domain of PTENβ is highlighted in the red box.

a

Accession	Gene	Function
O43660	PLRG1	mRNA splicing, via spliceosome
P52597	HNRNPF	nucleotide binding
Q9Y2X3	NOP58	biogenesis of box C/D snoRNAs such as U3, U8 and U14 snoRNAs
O00567	NOP56	biogenesis of box C/D snoRNAs such U3, U8 and U14 snoRNAs
P19338	NCL	pre-rRNA transcription and ribosome assembly
O15160	POLR1C	synthesize ribosomal RNA precursors
Q9Y2S0	POLR1D	synthesize ribosomal RNA precursors
F2Z3C0	RPS9	structural constituent of ribosome
M0R3D6	RPL18A	structural constituent of ribosome
G5E9W7	MRPS22	Ribonucleoprotein, Ribosomal protein
Q5JR95	RPS8	structural constituent of ribosome

b

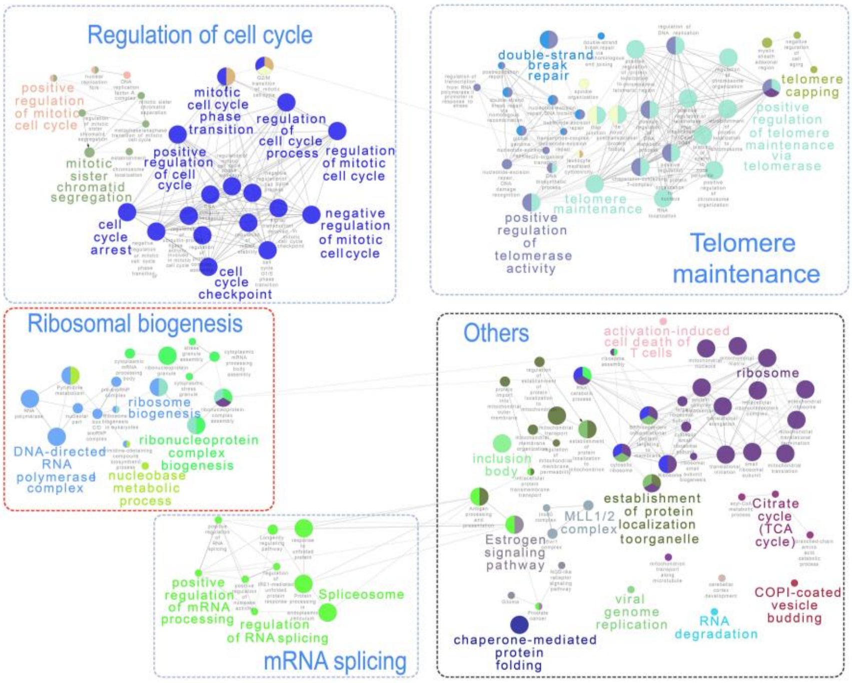

Supplementary Figure 4. Various potential PTENβ interacting nucleolar proteins identified by mass spectrometry analysis.

(**a**) Identification of nucleolar proteins that may interact with PTENβ by mass spectrometry analysis. Nucleolar proteins pull-downed by PTENβ which are more than 3 times as abundant as PTEN and PTENα control groups are listed.

(**b**) Functionally grouped KEGG and GO Biological Process and Cellular Component term annotation network of PTENβ pull-down genes. The nodes represent enriched terms, and node size indicates term enrichment significance after

Benjamini-Hochberg correction. The edges between two nodes represent initial genes shared between two enriched GO terms, and the thickness of the edges is based on their kappa scores. The calculated kappa score is also used for defining functional groups, which are displayed as nodes of the same color. The enriched terms of greatest interest are shown in bold face. The terms related to ribosomal biogenesis are highlighted in the red box. The terms related to additional ribosomal functions, such as the processing of mRNA, regulation of telomerase activity and regulation of the cell cycle are highlighted in blue boxes. The other terms are highlighted in the black box.

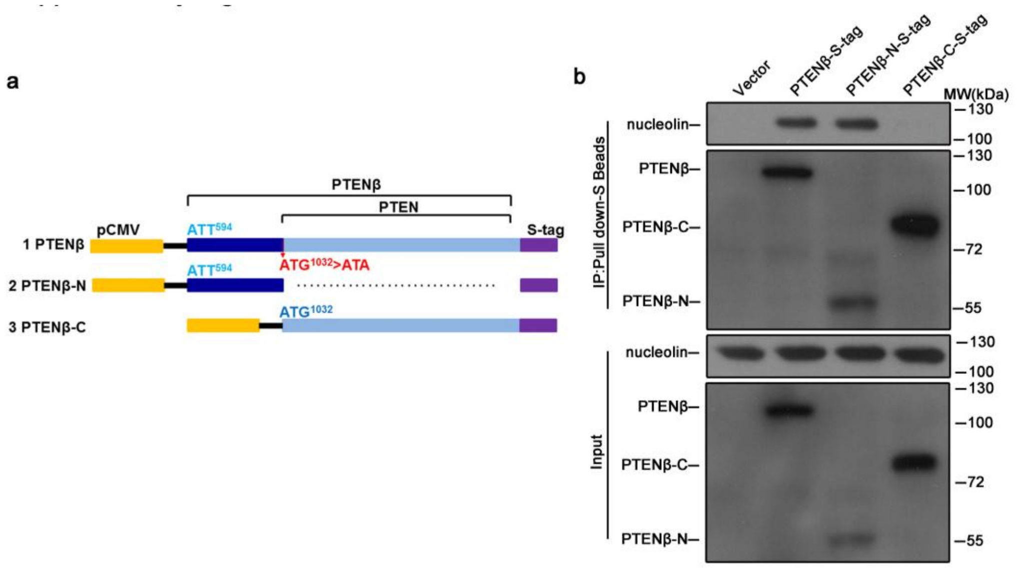

Supplementary Figure 5. The N-terminal domain of PTENβ is essential for its interaction with nucleolin.

(**a**) A set of full length or truncated PTENβ constructs with a C-terminal GFP tag. The AUG start codon of canonical PTEN was mutated to AUA in full length PTENβ constructs in order to abolish expression of canonical PTEN.

(**b**) The constructs indicated in (**a**) were introduced into HEK 293T cells, followed by immunoprecipitation with GFP antibody, followed by western blotting with an anti-nucleolin antibody.

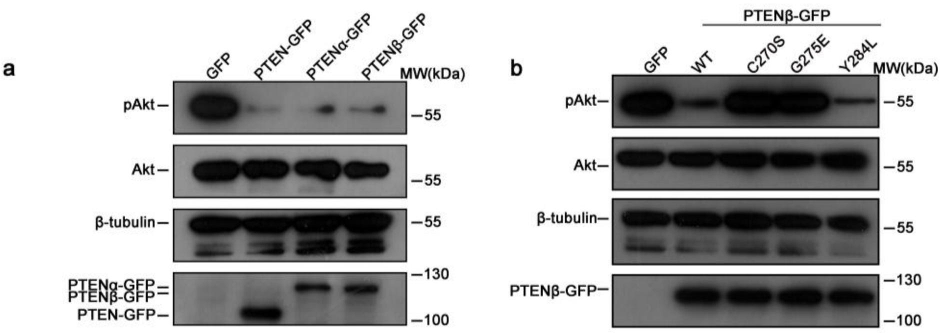

Supplementary Figure 6. PTENβ, like canonical PTEN, acts as an antagonist of the PI3K pathway.

(**a**) C-terminal GFP tagged PTEN, PTENα or PTENβ were separately introduced into 786-O cells (no endogenous PTEN expression) followed by immunoblotting with p-AKT , AKT, or GFP antibody.

(**b**) C-terminal GFP tagged wild type PTENβ, PTENβ with lipid phosphatase activity abolished (G275E, analogous to PTEN (G129E)), PTENβ with protein phosphatase activity abolished (Y284L, analogous to PTEN (Y138L)) and PTENβ with both lipid and protein phosphatase activity abolished (C270S, analogous to PTEN (C124S)) were introduced separately into 786-O cells, followed by immunoblotting with p-AKT, AKT, or GFP antibodies.

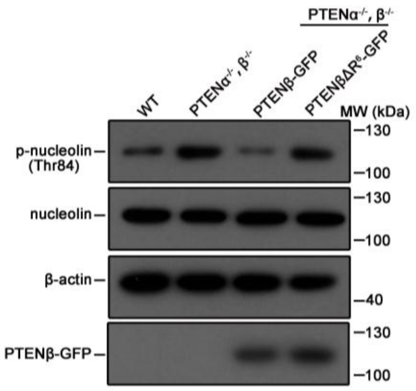

Supplementary Figure 7. The nucleolin phosphatase activity of PTENβ is linked to its localization in the nucleolus.

PTENα and PTENβ double knockout Hela cells were transfected with C-terminal GFP tagged PTENβ or C-terminal GFP tagged PTENβΔR^6, followed by immunoblotting with p-nucleolin (Thr 84), nucleolin, or GFP antibody.

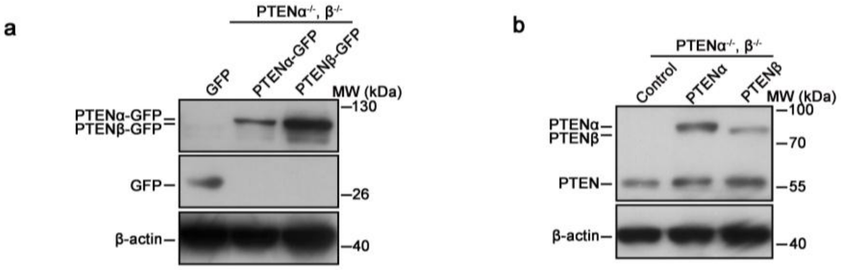

Supplementary Figure 8. Determination of transfection efficiency.

(**a**) C-terminal GFP tagged PTENα and PTENβ were introduced into PTENα and PTENβ double knockout cells, followed by immunoblotting with GFP antibody. β-actin was used as a control.

(**b**) Exogenous PTENα and PTENβ without a tag were introduced into PTENα and PTENβ double knockout cells, followed by immunoblotting with a PTEN monoclonal antibody. β-actin was used as a control.

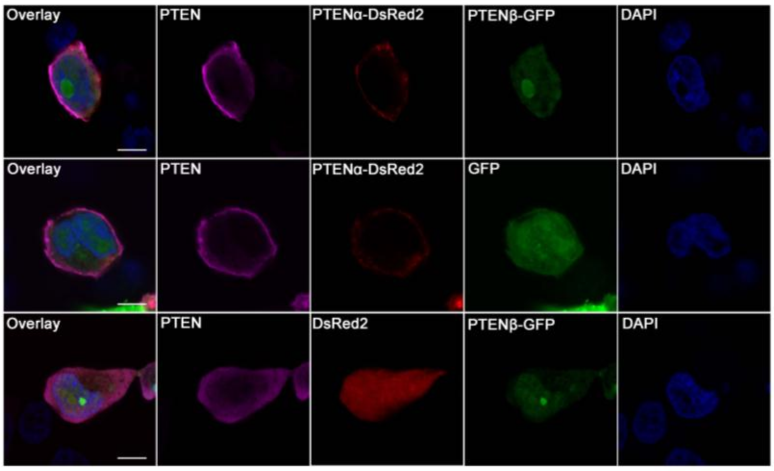

Supplementary Figure 9. The majority PTEN isoforms show mutually exclusive localization.

Plasmids expressing C-terminal DsRed2 tagged PTENα, C-terminal GFP tagged PTENβ and C-terminal FLAG tagged PTEN were constructed and co-transfected in Hela PTEN null cells. A monoclonal FLAG antibody (Sigma-Aldrich, F3165) was used to label PTEN. Empty PEGFP-N1 and pDsRed2-N1 plasmids were transfected as controls. The scale bars represent 5μm.

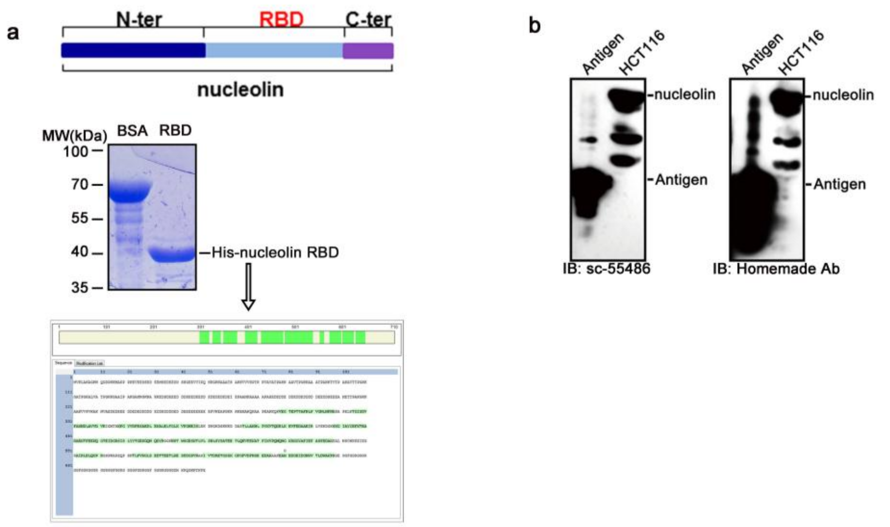

Supplementary Figure 10. Polyclonal anti-nucleolin antibody raised in our laboratory.

(**a**) The RNA binding domain of nucleolin (RBD) was cloned into the pET28a plasmid (upper panel) for protein purification and antibody generation. Purified His-RBD is shown in a SDS-PAGE gel (lower panel). Mass spectrometry analysis confirmed the peptide sequence of nucleolin.

(**b**) Verification of antibody specificity. Western blot analysis in HCT116 cells with nucleolin antibody established in our laboratory as indicated in (a), or with a commercial antibody (sc-55486).

a

```
LERGGEAAAAAAAAAAAPGRGSESPVTMSRAGNAGELVSPLLLPPTRRRRRRHIQGPGPV
LNLPSAAAAPPVARAPEAAGGGSRSEDYSSSPHSAAAAARPLAAEEKQAQSLQPSSSRRS
SHYPAAVQSQAAAERGASATAKSRAISILQKKPRHQQLLPSLSSFFFSHRLPDMTAIIKE
IVSRNKRRYQEDGFDLDLTYIYPNIIAMGFPAERLEGVYRNNIDDVVRFLDSKHKNHYKI
YNLCAERHYDTAKFNCRVAQYPFEDHNPPQLELIKPFCEDLDQWLSEDDNHVAAIHCKAG
KGRTGVMICAYLLHRGKFLKAQEALDFYGEVRTRDKKGVTIPSQRRYVYYYSYLLKNHLD
YRPVALLFHKMMFETIPMFSGGTCNPQFVVCQLKVKIYSSNSGPTRREDKFMYFEFPQPL
PVCGDIKVEFFHKQNKMLKKDKMFHFWVNTFFIPGPEETSEKVENGSLCDQEIDSICSIE
RADNDKEYLVLTLTKNDLDKANKDKANRYFSPNFKVKLYFTKTVEEPSNPEASSSTSVTP
DVSDNEPDHYRYSDTTDSDPENEPFDEDQHTQITKV
```

Human

b

```
LERGGEAAAAAAAPGRGSESPVTMARAGNAGELLSPLLLPPTRRRRRRHVQGPGPVLSLPSA
AAAPPLARAPEAAGGGSRCEDYPSSPHSAASAARPLAAEEKQAQSLQPSSSRRSSHYPAAVQ
GQAAAERGASATAKSRAISILQKKPRHQQLLPSLSSFFFSHRLPDMTAIIKEIVSRNKRRYQ
EDGFDLDLTYIYPNIIAMGFPAERLEGVYRNNIDDVVRFLDSKHKNHYKIYNLCAERHYDTA
KFNCRVAQYPFEDHNPPQLELIKPFCEDLDQWLSEDDNHVAAIHCKAGKGRTGVMICAYLLH
RGKFLKAQEALDFYGEVRTRDKKGVTIPSQRRYVYYYSYLLKNHLDYRPVALLFHKMMFETI
PMFSGGTCNPQFVVCQLKVKIYSSNSGPTRREDKFMYFEFPQPLPVCGDIKVEFFHKQNKML
KKDKMFHFWVNTFFIPGPEETSEKVENGSLCDQEIDSICSIERADNDKEYLVLTLTKNDLDK
ANKDKANRYFSPNFKVKLYFTKTVEEPSNPEASSSTSVTPDVSDNEPDHYRYSDTTDSDPEN
EPFDEDQHSQITKV
```

Mouse

Supplementary Figure 11. Sequences supplemented in the UniProt database.

(**a,b**) N-terminal extended PTEN sequence supplemented in the UniProt Human database (**a**) or in the UniProt Mouse database (**b**) for raw file searching by Proteome Discoverer. The most proximal N-terminal amino acids of PTENα or PTENβ are highlighted separately in green or red.

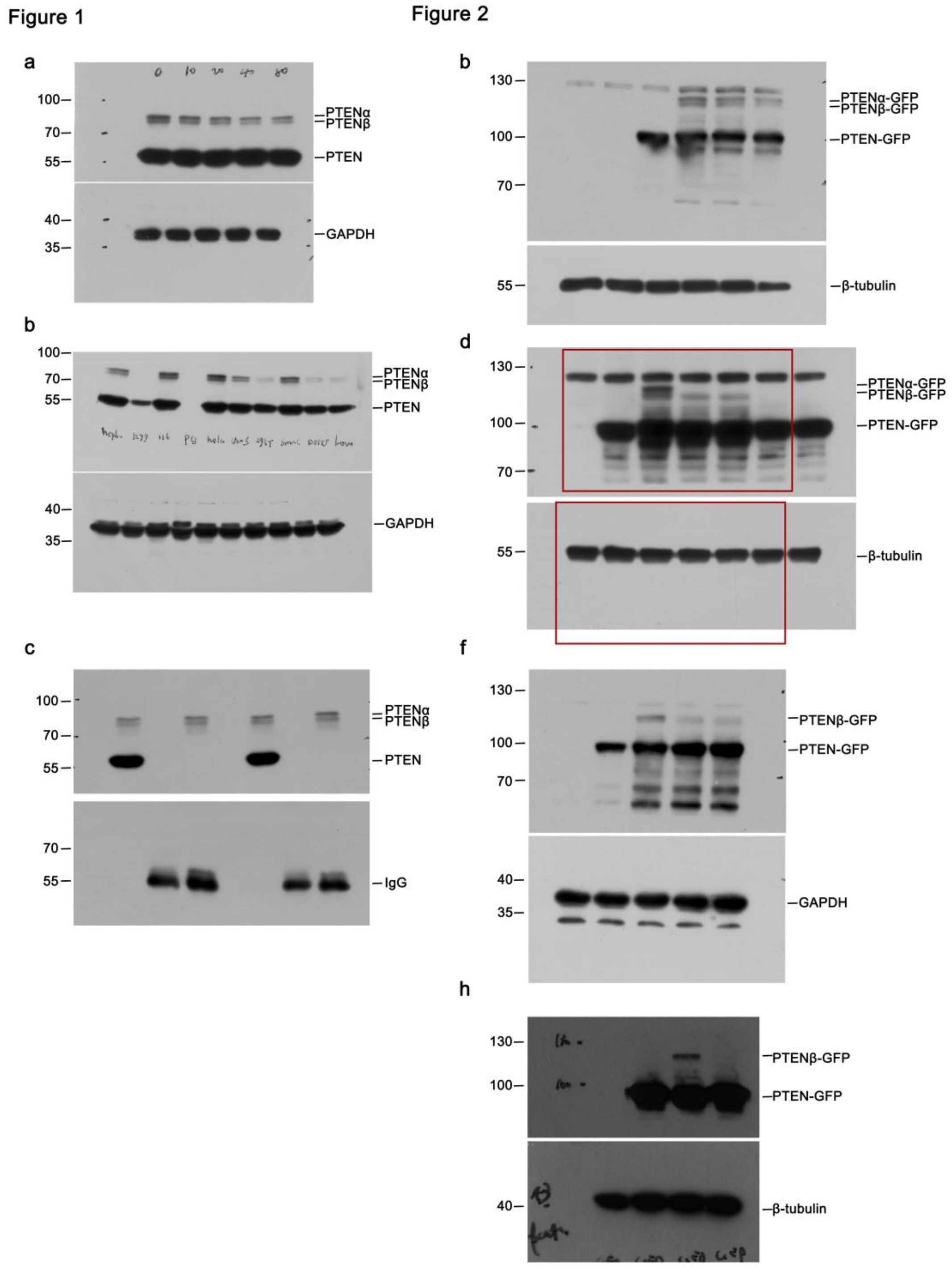

Supplementary Figure 12. Original immunoblots for indicated figures.

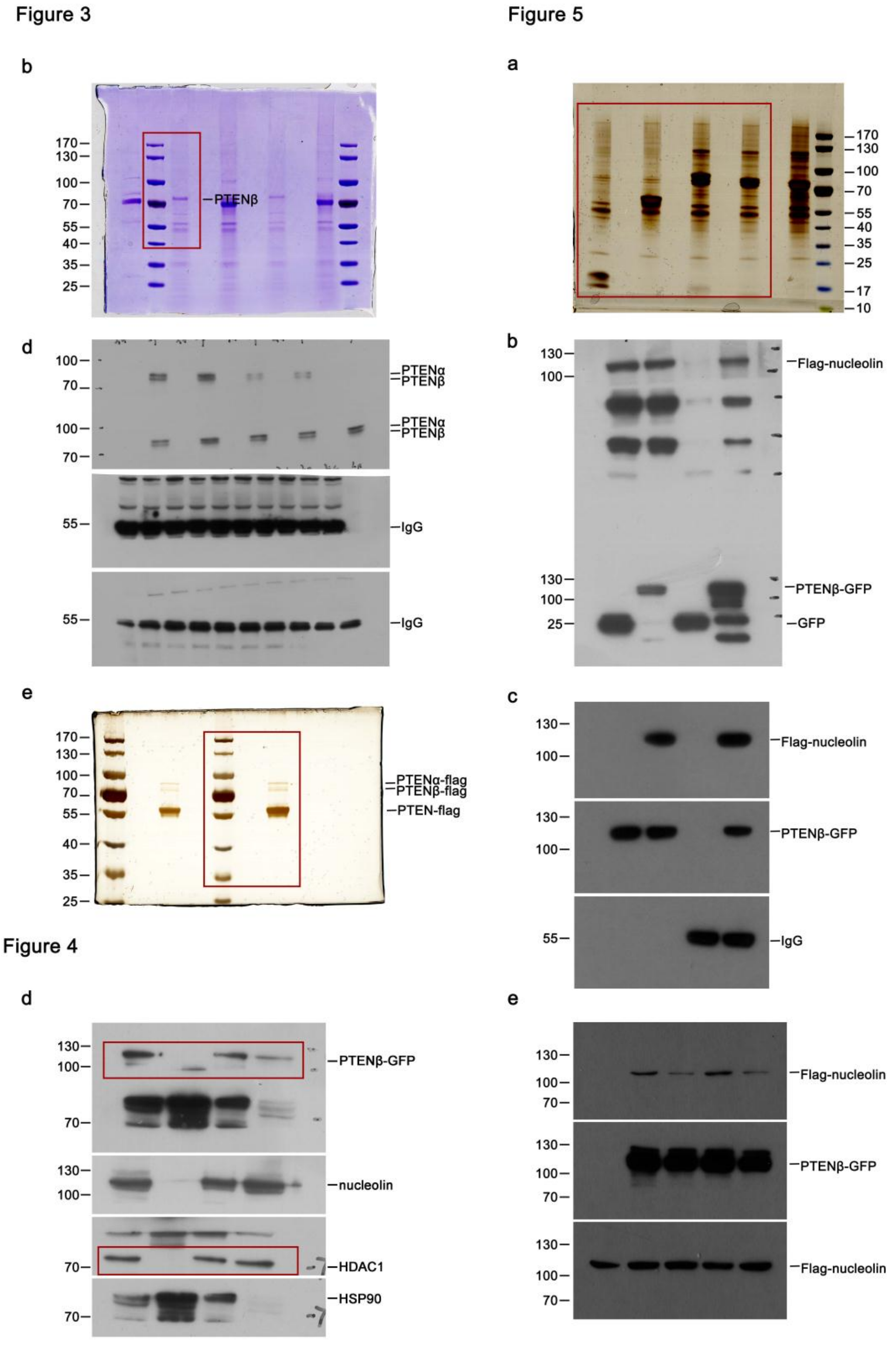

Supplementary Figure 13. Original immunoblots and polyacrylamide gels for indicated figures.

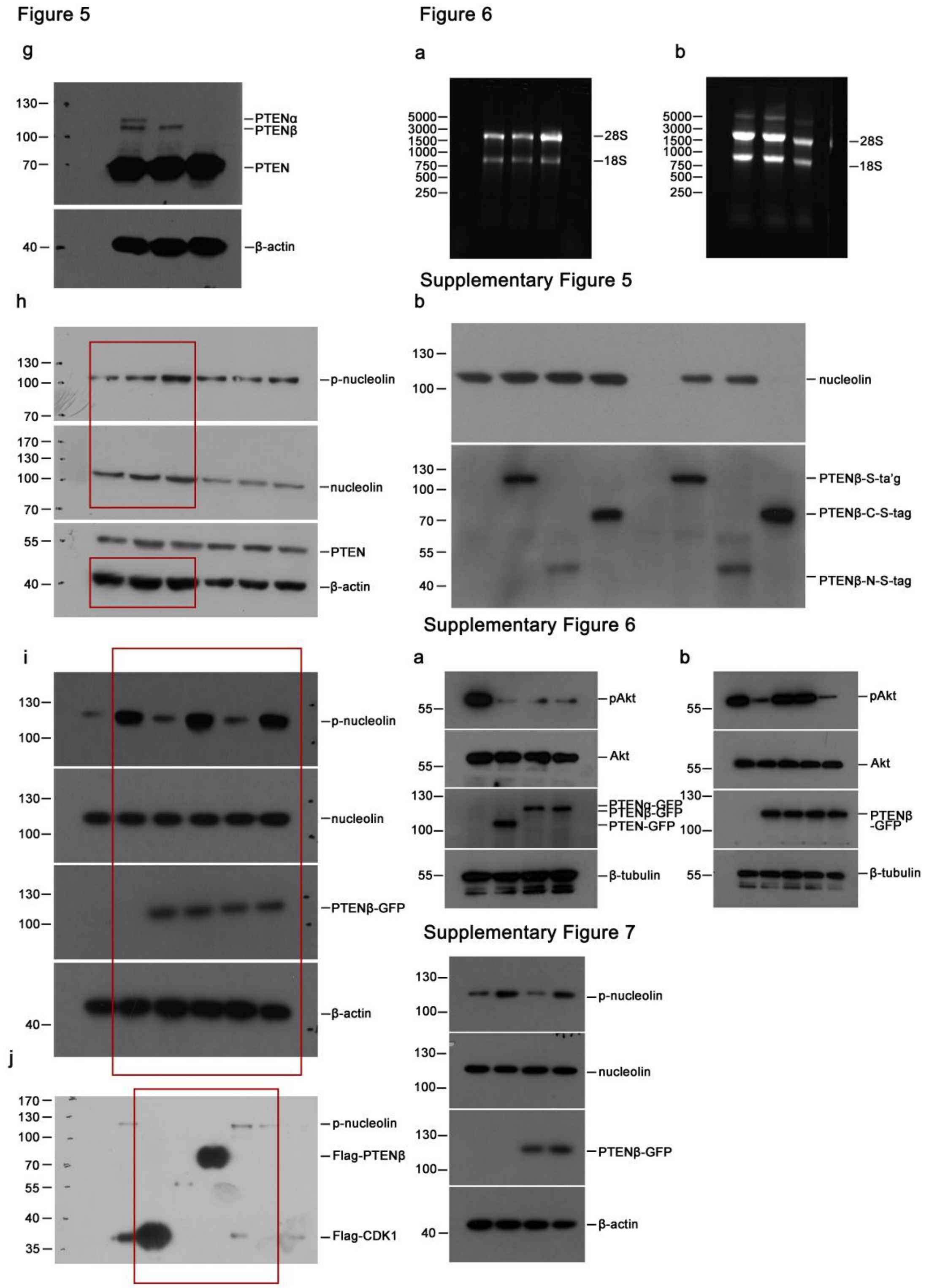

Supplementary Figure 14. Original immunoblots and agarose gels for indicated figures.

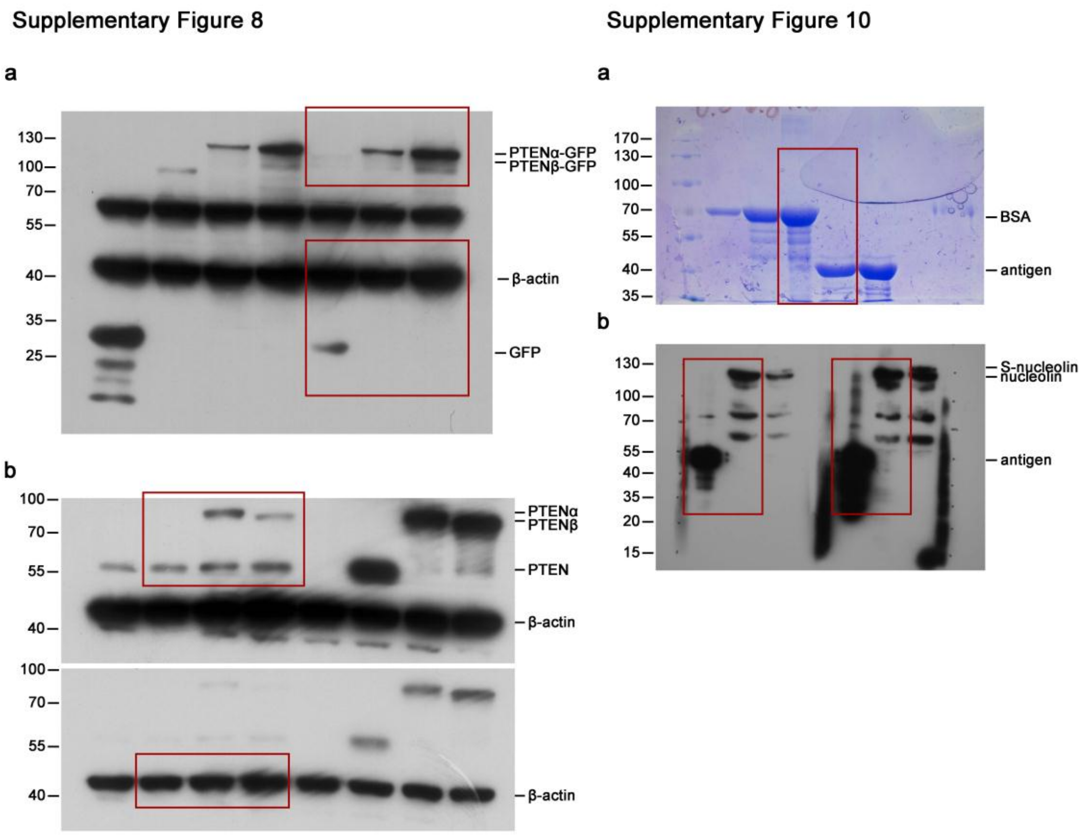

Supplementary Figure 15. Original immunoblots and polyacrylamide gels for indicated figures.

Supplementary Table 1. A list of antibodies used in this study

Species	Antigen	Clone#	Company (Cat#)	Dilution ratio			Citation
				Immuno blotting	Immuno precipitation	Immuno fluorescence	
Mouse monoclonal	PTEN	A2B1	Santa Cruz (sc-7974)	1:1000		1:200	1
Rabbit monoclonal	PTEN	138G6	Cell Signaling (#9559)	1:1000			2
Mouse monoclonal	PTEN	6H2.1	ABM (#2502)			1:200	3
Rabbit polyclonal	GFP		MBL (#598)	1:5000	1:500		4
Rabbit polyclonal	AKT		Cell Signaling (#9272)	1:1000			5
Rabbit polyclonal	AKT Phospho (T473)		Cell Signaling (#9271)	1:1000			5
Rabbit monoclonal	nucleolin Phospho (T84)	EPR8080	Abcam (ab155977)	1:2000			N/A
Mouse monoclonal	UBF	F-9	Santa Cruz (sc-13125)			1:200	6
Rabbit polyclonal	β-actin		MBL (pm053)	1:5000			7
Mouse monoclonal	GAPDH	1C4	Sungene Biotech (KM9002)	1:5000			N/A
Mouse monoclonal	FLAG	M2	Sigma-Aldrich (F3165)	1:5000	1:500	1:300	8
Mouse monoclonal	β-tubulin	3G7	Sungene Biotech (KM9003)	1:5000			N/A
Mouse polyclonal	PTENα		Homemade		1:500		9
Rabbit polyclonal	nucleolin		Homemade	1:2000			

Supplementary Table 2. Primers for constructing plasmids

Vector	Forward primer (5’-3’)	Backward primer (5’-3’)
PTEN-GFP	CCGGAATTCATGACAGCCATCATCAAAGAG	CGCGGATCCGCGACTTTTGTAATTTGTGTATGC
PTENα-GFP	CCGGAATTCCTGGAGCGGGGGGGAGAAG	CGCGGATCCGCGACTTTTGTAATTTGTGTATGC
PTENβ-GFP	CCGGAATTCATTTCCAGGGCTGGGAACG	CGCGGATCCGCGACTTTTGTAATTTGTGTATGC
PTEN-S-tag	CCGGAATTCATGACAGCCATCATCAAAGAG	CGCGGATCCGACTTTTGTAATTTGTGTATGC
PTENα-S-tag	CCGGAATTCCTGGAGCGGGGGGGAGAAG	CGCGGATCCGACTTTTGTAATTTGTGTATGC
PTENβ-S-tag	CCGGAATTCATTTCCAGGGCTGGGAACG	CGCGGATCCGACTTTTGTAATTTGTGTATGC
PTENα-His	CCGGAATTCCTGGAGCGGGGGGGAGAAG	CGCGGATCCGACTTTTGTAATTTGTGTATGC
FLAG-nucleolin	CGCGGATCCATGGTGAAGCTCGCGAAGGC	CCGGAATTCCTATTCAAACTTCGTCTTC

Supplementary Table 3. Primers for mutagenesis

Vector	Forward primer (5’-3’)	Backward primer (5’-3’)
PTENα CTG^{513}>CTC	CGGCACCTCCCGCTCCTCGAGCGGGGGGGAGAAG	CTTCTCCCCCCCGCTCGAGGAGCGGGAGGTGCCG
PTENα ATT^{594}>CTC	GAGTCGCCTGTCACCCTCTCCAGGGCTGGGAAC	GTTCCCAGCCCTGGAGAGGGTGACAGGCGACTC
PTENα TTG^{621}>CTC	GGGAACGCCGGAGAGCTCGTCTCTCCCCTTCTA	TAGAAGGGGAGAGACGAGCTCTCCGGCGTTCCC
PTENα ATG^{1032}>ATA	CACAGGCTCCCAGACATAACAGCCATCATCAAAG	CTTTGATGATGGCTGTTATGTCTGGGAGCCTGTG
PTENβ ACC^{591} deletion	TCTGAGTCGCCTGTCATTTCCAGGGCTTAG	CTAAGCCCTGGAAATGACAGGCGACTCAGA
PTENβ GTCACC^{588} deletion	GGGTCTGAGTCGCCTATTTCCAGGGCTGGG	CCCAGCCCTGGAAATAGGCGACTCAGACCC
PTENβ TGT^{372}>TCT	TTGCAGCAATTCACTCTAAAGCTGGAAAGGGAC	GTCCCTTTCCAGCTTTAGAGTGAATTGCTGCAA
PTENβ GGA^{387}>GAA	GTAAAGCTGGAAAGGAACGAACTGGTGTAATG	CATTACACCAGTTCGTTCCTTTCCAGCTTTAC
PTENβ TAT^{414}>CTT	GTAATGATATGTGCACTTTTATTACATCGGGGC	GCCCCGATGTAATAAAAGTGCACATATCATTAC
PTENβ ATT^{594} downstream palindrome disruption	GAACGCCGGAGAATTGGTCTCAGCCCTTCTACTGCC	GGCAGTAGAAGGGCTGAGACCAATTCTCCGGCGTTC

Supplementary Table 4. Primers for RT-PCR

Target	Forward primer (5'-3')	Backward primer (5'-3')
45S pre-rRNA	CCTGCTGTTCTCTCGCGCG TCCGAG	AACGCCTGACACGCACGGC ACGGAG

Supplementary References

1. Zhang H, Zhou X, Xu C, Yang J, Xiang J, Tao M*, et al.* Synergistic tumor suppression by adenovirus-mediated ING4/PTEN double gene therapy for gastric cancer. *Cancer gene therapy* 2016, **23**(1)**:** 13-23.
2. Wan X, Helman LJ. Levels of PTEN protein modulate Akt phosphorylation on serine 473, but not on threonine 308, in IGF-II-overexpressing rhabdomyosarcomas cells. *Oncogene* 2003, **22**(50)**:** 8205-8211.
3. Kwak MK, Johnson DT, Zhu C, Lee SH, Ye DW, Luong R*, et al.* Conditional deletion of the Pten gene in the mouse prostate induces prostatic intraepithelial neoplasms at early ages but a slow progression to prostate tumors. *PloS one* 2013, **8**(1)**:** e53476.
4. Tsuruta F, Green EM, Rousset M, Dolmetsch RE. PIKfyve regulates CaV1.2 degradation and prevents excitotoxic cell death. *The Journal of cell biology* 2009, **187**(2)**:** 279-294.
5. Franke TF, Kaplan DR, Cantley LC. PI3K: downstream AKTion blocks apoptosis. *Cell* 1997, **88**(4)**:** 435-437.
6. Wandrey F, Montellese C, Koos K, Badertscher L, Bammert L, Cook AG*, et al.* The NF45/NF90 Heterodimer Contributes to the Biogenesis of 60S Ribosomal Subunits and Influences Nucleolar Morphology. *Molecular and cellular biology* 2015, **35**(20)**:** 3491-3503.
7. Wang G, Li Y, Wang P, Liang H, Cui M, Zhu M*, et al.* PTEN regulates RPA1 and protects DNA replication forks. *Cell research* 2015, **25**(11)**:** 1189-1204.
8. Gu X, Mao X, Lussier MP, Hutchison MA, Zhou L, Hamra FK*, et al.* GSG1L suppresses AMPA receptor-mediated synaptic transmission and uniquely modulates AMPA receptor kinetics in hippocampal neurons. *Nature communications* 2016, **7:** 10873.
9. Liang H, He S, Yang J, Jia X, Wang P, Chen X*, et al.* PTENalpha, a PTEN isoform translated through alternative initiation, regulates mitochondrial function and energy metabolism. *Cell metabolism* 2014, **19**(5)**:** 836-848.
